# Sodium-Glucose Linked Transporter-2 (SGLT-2) Inhibitor-Associated Unmasking of Myasthenia Gravis in a Patient With Undiagnosed Thymic Follicular Hyperplasia: The First Reported Case

**DOI:** 10.7759/cureus.110375

**Published:** 2026-06-06

**Authors:** Asad Waqar, Okasha Zulfiqar, Favour K Mbakwe, Talha Ali, Ghulam Mohayy Ud Din

**Affiliations:** 1 Acute Medicine, Bradford Teaching Hospitals NHS Foundation Trust, Bradford, GBR; 2 Internal Medicine, Mayo Hospital, Lahore, PAK; 3 Internal Medicine, Obafemi Awolowo University, Ile-Ife, NGA; 4 Cardiac Surgery, Mayo Hospital, Lahore, PAK

**Keywords:** drug-induced autoimmunity, empagliflozin, myasthenia gravis, sglt-2 inhibitor, thymic hyperplasia

## Abstract

Myasthenia gravis is an autoimmune disease of the neuromuscular junction, primarily caused by antibodies directed against the acetylcholine receptor. Follicular hyperplasia of the thymus can be seen in patients with acetylcholine receptor-positive myasthenia gravis and may remain asymptomatic for many years until unmasked by a specific medication. Myasthenia gravis induced by drugs has been reported with D-penicillamine and immune checkpoint inhibitors, but never with the sodium-glucose linked transporter-2 inhibitors. We report a case of a 62-year-old man with type 2 diabetes presenting with progressive, fluctuating bilateral ptosis, binocular diplopia, dysarthria, and dysphagia occurring eight weeks after starting treatment with 10 mg daily of empagliflozin. He had no history of any other medications or infections. Physical examination revealed fluctuating bilateral ptosis (more prominent on the right) with an ice pack test demonstrating a 2 mm improvement after two minutes. The anti-acetylcholine receptor antibody level was positive at 4.2 nmol/L. Repetitive nerve stimulation studies revealed an 18% decremental response at 3 Hz. A chest computed tomography scan showed diffuse bilateral symmetric thymic hyperplasia measuring 12 mm in size without any mass. Empagliflozin was discontinued. Pyridostigmine and prednisone 40 mg daily were started, with full resolution of his symptoms. The patient shows no symptoms at one year on pyridostigmine alone. This case represents the first description of the unmasking of acetylcholine receptor-positive myasthenia gravis by sodium-glucose linked transporter-2 inhibitors in a patient with thymic follicular hyperplasia. Myasthenia gravis should be considered in patients with newly acquired fatigable ptosis, diplopia, or bulbar symptoms who are being treated with sodium-glucose linked transporter-2 inhibitors.

## Introduction

Myasthenia gravis (MG) is a type of autoimmune neuromuscular junction disorder characterized by fluctuating skeletal muscle weakness and fatigue [1]. MG usually involves autoantibody responses to the acetylcholine receptor (AChR), leading to neuromuscular transmission failure due to complement-mediated destruction, degradation, and blockade of receptors [2]. MG is commonly diagnosed based on symptoms such as ptosis, diplopia, and bulbar signs [1,2]. In almost all cases, MG patients experience generalized symptoms within two years of onset [1,2]. The thymus gland is essential in the development of AChR-positive MG due to its ability to produce autoimmune sensitization [3]. Follicular hyperplasia of the thymus, common among MG patients, results in the generation of high-affinity anti-AChR antibodies [4]. Usually, thymic follicular hyperplasia remains asymptomatic as subclinical disease until an additional factor triggers immune dysregulation and leads to clinical disease, as described in cases of latent MG precipitated by trauma or other triggers [4].

The use of sodium-glucose linked transporter-2 (SGLT-2) inhibitors in the management of type 2 diabetes mellitus has gained popularity due to their cardiovascular and renal benefits [5,6]. Although their side effect profile is relatively low, there is increasing evidence that SGLT-2 inhibitors can cause some immunomodulation, including alterations in T-cell activity and inflammation [6]. Immunologic alterations caused by SGLT-2 inhibitors might have unexpected implications for patients who are genetically susceptible to developing an autoimmune disorder [5,6]. Drug-induced or drug-unmasked MG is a known entity, usually triggered by drugs like D-penicillamine and immune checkpoint inhibitors (ICIs) [7]. No documented literature exists to date correlating SGLT-2 inhibitors with the development of MG [6-9].

We describe a patient with pre-existing but unrecognized thymic follicular hyperplasia who developed MG positive for antibodies to AChR shortly after the initiation of empagliflozin. Our findings imply a possible link between SGLT-2 inhibitors and the unveiling of an underlying MG.

## Case presentation

A 62-year-old Asian man with a 10-year history of type 2 diabetes mellitus (managed with metformin 1000 mg twice daily and sitagliptin 100 mg daily) and well-controlled hypertension (on lisinopril 10 mg daily) presented with a four-week history of fluctuating double vision and drooping eyelids. He had been started on empagliflozin 10 mg daily eight weeks prior to symptom onset and was also taking tamsulosin 0.4 mg daily for benign prostatic hyperplasia.

His symptoms began gradually, starting with intermittent right eyelid drooping, initially attributed to fatigue. Over two weeks, ptosis became bilateral (right greater than left) and was accompanied by horizontal diplopia, especially during reading or watching television. He later developed difficulty swallowing solid foods, requiring smaller bites and increased water intake. His voice became nasal, and all symptoms worsened throughout the day and with sustained upward gaze. He denied shortness of breath, limb weakness, or sensory symptoms.

Eight weeks before symptom onset, empagliflozin 10 mg daily was added to his regimen of metformin 1000 mg twice daily and sitagliptin 100 mg daily to improve glycemic control (HbA1c: 7.8%). No other new medications, vaccines, or infections were reported in the prior six months. He also took lisinopril 10 mg daily for hypertension and tamsulosin 0.4 mg daily for benign prostatic hyperplasia, both stable for years. He had no history of autoimmune or neurologic disorders, and his family history was negative for MG, thymoma, or other autoimmune diseases. He was a former smoker (quit 15 years ago; 20-pack-year history) and a retired accountant living with his spouse.

**Figure 1:**
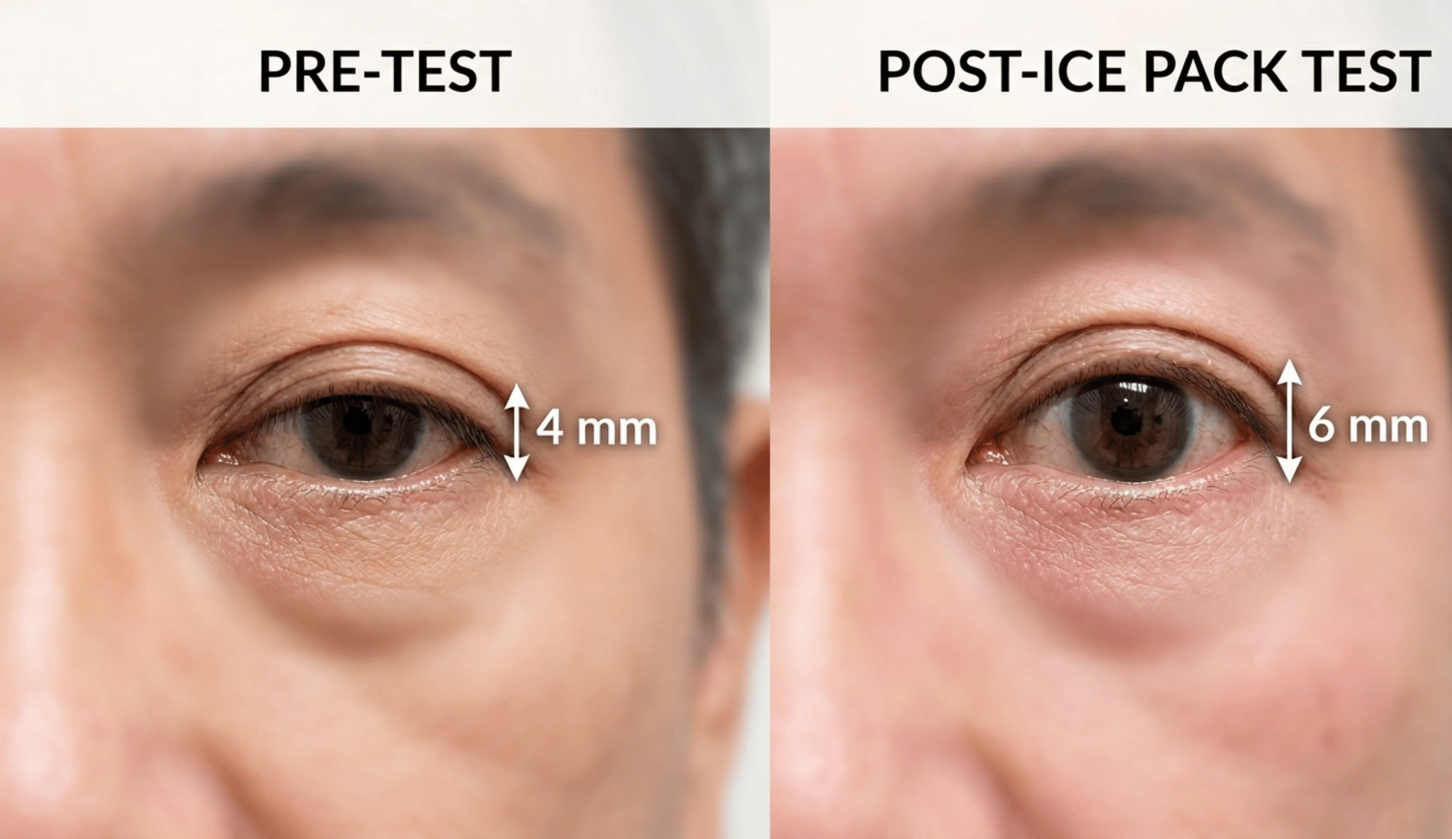
Representative photographs of the ice pack test. The baseline right palpebral aperture of 4 mm (left) improved to 6 mm (right) after the procedure, supporting a diagnosis of myasthenia gravis

On examination, the patient was alert, oriented, and in no acute distress. Vital signs were normal: blood pressure 128/78 mmHg, heart rate 72 beats per minute and regular, respiratory rate 14 breaths per minute, and afebrile. The cranial nerve exam showed bilateral ptosis with a right palpebral aperture of 4 mm and a left of 6 mm at baseline. After 60 seconds of upward gaze, ptosis worsened to 2 mm on the right and 4 mm on the left. Extraocular movements were limited in abduction and elevation bilaterally, with diplopia on both left and right gazes. The ice pack test improved the right palpebral aperture from 4 mm to 6 mm, which is positive for MG (Figure 1).

Pupils were equal, round, and reactive to light and accommodation, with no relative afferent pupillary defect. The bulbar exam showed mild nasal dysarthria, weak palatal elevation, and a diminished but present gag reflex bilaterally. The motor exam revealed 5/5 strength in all extremities, with no fatigable weakness on repetitive hand grip or arm abduction. Deep tendon reflexes were 2+ and symmetric, with flexor plantar responses. Sensation, coordination, and gait were normal.

**Figure 2:**
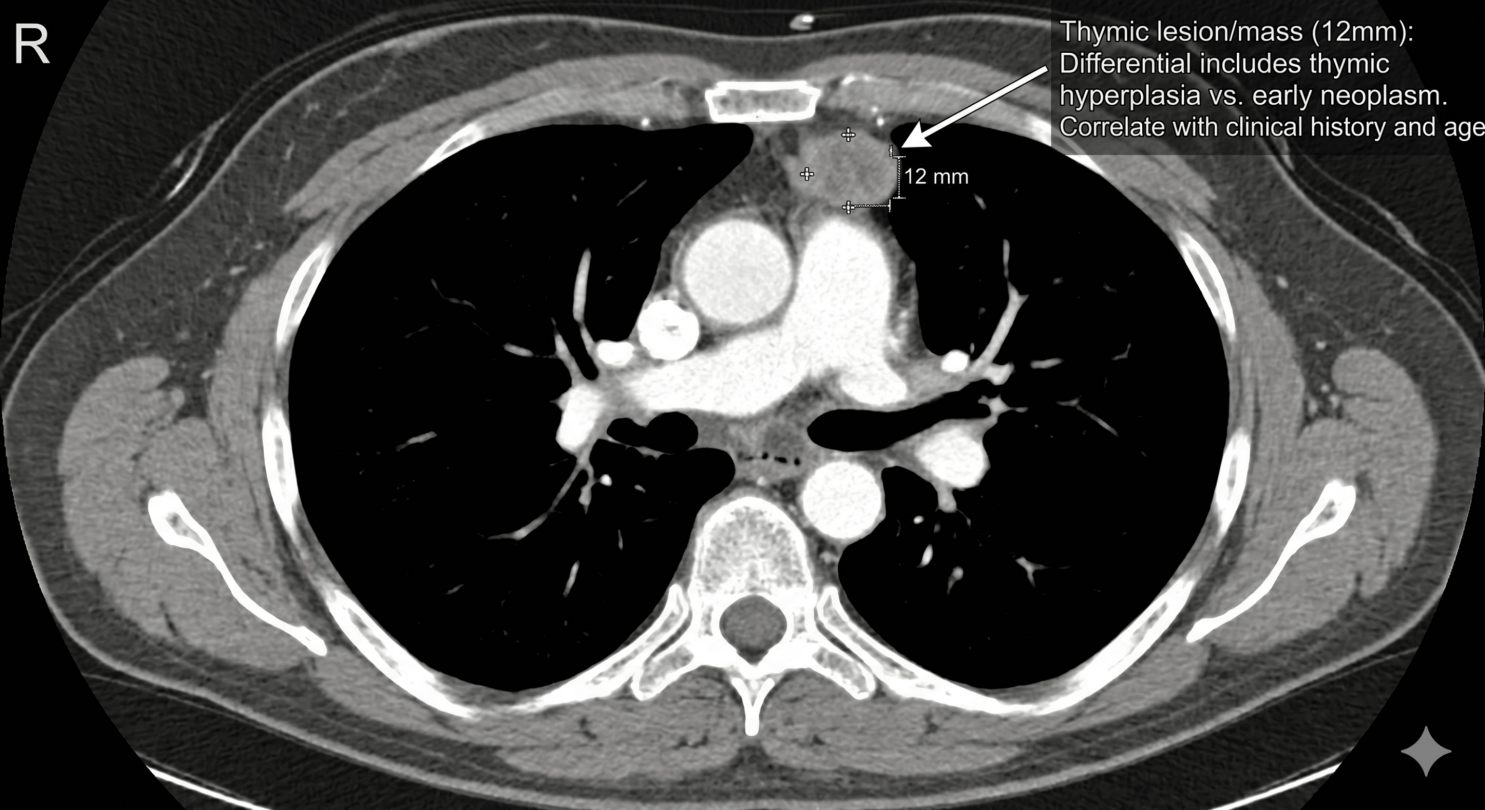
Chest computed tomography scan showing thymic hyperplasia in the anterior mediastinum Contrast-enhanced computed tomography (portal venous phase, slice thickness 2.5 mm) of the chest demonstrates diffuse, symmetric enlargement of the thymus measuring 12 mm in maximum thickness, without discrete nodules or invasive features.

Diagnostic assessment confirmed MG. AChR-binding antibodies were elevated at 4.2 nmol/L (reference: <0.4 nmol/L), while anti-muscle-specific kinase (anti-MuSK) and anti-lipoprotein receptor-related protein 4 (anti-LRP4) antibodies were negative. Additional AChR antibody testing showed modulating antibodies with 35% loss (reference: <20% loss) and blocking antibodies with 15% block (reference: <5% block), both positive. Creatine kinase was normal at 85 U/L, excluding myopathy. Repetitive nerve stimulation of the facial nerve at 3 Hz showed an 18% decremental response, confirming a postsynaptic neuromuscular junction defect. Single-fiber electromyography of the frontalis muscle demonstrated increased jitter in 14 of 20 fiber pairs (70%), further supporting the diagnosis of MG. Figure 2 shows the chest computed tomography with intravenous contrast, revealing diffuse, symmetric thymic enlargement measuring 12 mm in maximum thickness without discrete nodules or invasive features, findings suggestive of thymic hyperplasia. The definitive diagnosis of thymic follicular hyperplasia was later confirmed by histopathological examination following thymectomy.

A summary of baseline laboratory and neurophysiological tests is provided in Table 1.

**Table 1:** Summary of laboratory and neurophysiological investigations MG: myasthenia gravis; AChR: acetylcholine receptor; MuSK: muscle-specific kinase; LRP4: lipoprotein receptor-related protein 4; ANA: antinuclear antibody; SLE: systemic lupus erythematosus; CK: creatine kinase; ESR: erythrocyte sedimentation rate; CRP: C-reactive protein; EMG: electromyography; Hz: hertz

Category	Test	Result	Reference range	Interpretation
Hematology	Hemoglobin	14.2 g/dL	13.5-17.5 g/dL	Normal
	White blood cell count	6.8×10³/μL	4.0-11.0×10³/μL	Normal
	Platelet count	245×10³/μL	150-450×10³/μL	Normal
Metabolic	Fasting glucose	145 mg/dL	70-100 mg/dL	Elevated
	HbA1c	7.2%	<5.7%	Elevated
MG-specific autoantibodies	Anti-AChR (binding)	4.2 nmol/L	<0.4 nmol/L	Positive: diagnostic of MG
	Anti-AChR (modulating)	35% loss	<20% loss	Positive
	Anti-AChR (blocking)	15% block	<5% block	Positive
	Anti-MuSK	Negative	Negative	Normal: rules out MuSK-MG
	Anti-LRP4	Negative	Negative	Normal
Other autoantibodies	ANA	Negative	<1:80	Normal: no SLE
	Rheumatoid factor	<10 IU/mL	<14 IU/mL	Normal
Muscle enzymes	CK	85 U/L	30-200 U/L	Normal: no myopathy
Inflammatory markers	ESR	12 mm/hr	0-22 mm/hr	Normal
	CRP	2.1 mg/L	<5.0 mg/L	Normal
Neurophysiology	Repetitive nerve stimulation (3 Hz, facial nerve)	18% decrement	<10%	Abnormal: postsynaptic defect
	Single-fiber EMG (frontalis)	14/20 pairs with jitter (70%)	<10% abnormal	Abnormal: highly sensitive for MG

**Figure 3:**
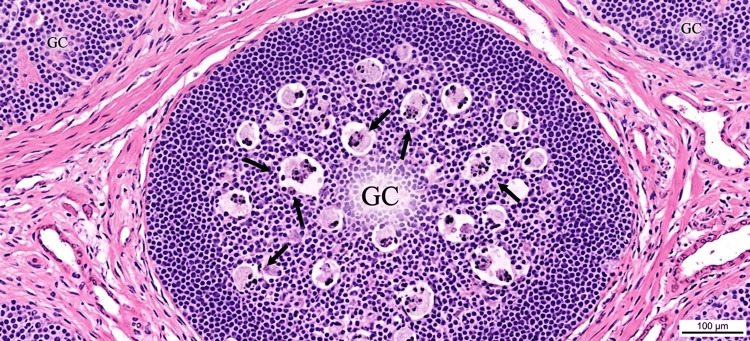
Follicular thymic hyperplasia with prominent germinal centers Histopathological examination (H&E, scale bar: 100 micrometers). The micrograph shows multiple large lymphoid follicles (follicular hyperplasia) embedded in a background of fibrous stroma. A prominent GC is highly visible, characterized by a central zone of pale-staining cells. Within the GC, multiple tingible-body macrophages (indicated by the black arrows) are present, filled with characteristic clear cytoplasmic spaces and engulfed apoptotic debris, indicating a robust and active follicular immune response. GC: germinal center

Empagliflozin was discontinued as the suspected trigger. Pyridostigmine 60 mg every six hours was started, resulting in partial improvement in ptosis and swallowing within 48 hours. Prednisone 40 mg daily (approximately 0.5 mg/kg) was added and tapered over 12 weeks. Video-assisted thoracoscopic thymectomy was performed at three months. The thymus weighed 38 grams (normal adult weight: 20-30 grams), and histopathology showed follicular thymic hyperplasia with prominent germinal centers containing tingible-body macrophages (Figure 3).

At the 12-month follow-up, the patient remains asymptomatic on pyridostigmine 60 mg three times daily. Prednisone has been discontinued without relapse. His MG Foundation of America post-intervention status is pharmacologic remission [8], and his diabetes is controlled on metformin, sitagliptin, and low-dose insulin glargine (HbA1c: 7.0%). The patient's perspective is provided in the Patient Perspective section. Table 2 shows the clinical course from presentation to resolution of symptoms.

**Table 2:** Timeline of clinical events HbA1c: glycated hemoglobin; R>L: right greater than left; q6h: every 6 hours; VATS: video-assisted thoracoscopic surgery; TID: three times daily

Time point	Event
8 weeks prior	Empagliflozin 10 mg daily started (baseline HbA1c 7.8%)
4 weeks prior	Intermittent right ptosis, worse in evenings
3 weeks prior	Bilateral ptosis (R>L); horizontal diplopia begins
2 weeks prior	Dysphagia to solids; nasal voice
Presentation day	Neurology evaluation and diagnostic workup
Day 1	Empagliflozin stopped; pyridostigmine 60 mg q6h started
Day 7	~50% ptosis improvement; prednisone 40 mg daily added
Week 4	Ptosis resolved at rest; mild diplopia on extreme gaze
Week 12	Prednisone tapered; stable on pyridostigmine alone
Month 3	VATS thymectomy; follicular hyperplasia confirmed
Month 12	Asymptomatic; on pyridostigmine 60 mg TID

## Discussion

We describe the first reported case of MG unmasked by an SGLT‑2 inhibitor (empagliflozin) in a patient with prior asymptomatic thymic hyperplasia. To substantiate the novelty of this finding, we conducted a systematic literature search on PubMed and Google Scholar (last search: May 2026, no language restrictions) using the following terms: (“SGLT‑2 inhibitor” OR “empagliflozin” OR “dapagliflozin” OR “canagliflozin” OR “ertugliflozin”) AND (“myasthenia gravis” OR “acetylcholine receptor antibody” OR “neuromuscular junction”). We also reviewed the Food and Drug Administration (FDA) Adverse Event Reporting System (FAERS) and the World Health Organization (WHO) VigiBase for reported cases of MG associated with SGLT‑2 inhibitors. No previous reports of SGLT‑2 inhibitor‑associated MG were identified. Thus, this case represents the first such description.

The time course (symptom onset eight weeks after drug initiation), absence of other triggers (no new medications, vaccines, or infections), positive dechallenge (improvement after empagliflozin discontinuation), confirmatory testing (AChR antibodies 4.2 nmol/L, 18% decrement on repetitive nerve stimulation), and histopathology (thymic follicular hyperplasia, 38 g) support a causal link. The Naranjo Adverse Drug Reaction Probability Scale score of 6 indicates a probable adverse drug reaction [4].

To contextualize our findings, we reviewed the literature on drug-induced or drug-unmasked MG. Mouratidou et al. reported a case of pembrolizumab‑induced MG in a 71‑year‑old man with lung cancer [5]. The patient developed diaphragmatic weakness and respiratory symptoms six weeks after starting ICI therapy, with elevated AChR antibodies [5]. However, the mechanistic basis of ICI‑associated MG is fundamentally different from the hypothesized mechanism in our case. ICIs directly block PD‑1/CTLA‑4 immune checkpoints, leading to T‑cell disinhibition and well‑characterized autoimmune toxicities. In contrast, SGLT‑2 inhibitors have no known direct checkpoint effects; their immunomodulatory actions include anti‑inflammatory effects such as NLRP3 inflammasome attenuation and macrophage M2 polarization [10]. Although a paradoxical unmasking of subclinical autoimmunity might occur in susceptible individuals, the level of evidence supporting SGLT‑2 inhibitors as autoimmune triggers is not equivalent to that for ICIs. Therefore, we do not claim equivalence but rather generate a hypothesis that SGLT‑2 inhibitors may unmask latent MG in patients with pre‑existing thymic pathology. Confirmation awaits further pharmacovigilance and mechanistic studies.

The latency period of six weeks in that case is similar to the eight-week onset observed in our patient [5]. Both cases demonstrate that immunomodulatory therapies, whether ICIs or SGLT-2 inhibitors, can unmask subclinical MG in susceptible individuals [5]. However, our patient had a milder clinical course limited to ocular and bulbar symptoms without respiratory involvement, whereas the pembrolizumab-induced case presented with life-threatening diaphragmatic weakness, highlighting the variable expressivity of drug-unmasked MG [5].

Moreover, Feng et al. reported a case of dapagliflozin-associated bullous pemphigoid, an autoimmune blistering disorder, in a patient with type 2 diabetes [6]. The patient developed tense bullae on the trunk and extremities several months after initiating dapagliflozin, with histopathology confirming subepidermal blistering and linear deposition of IgG and C3 along the basement membrane [6]. The condition improved after the discontinuation of the SGLT-2 inhibitor and the initiation of topical corticosteroids [6]. This case further supports the concept that SGLT-2 inhibitors can trigger autoimmune events in susceptible individuals, extending beyond neuromuscular disorders to cutaneous autoimmunity [6].

The D-penicillamine-induced MG described by Antos et al. contrasts clearly with our case. In their patient, a 51‑year‑old man with Wilson's disease developed diplopia and evening ptosis after 15 months of D‑penicillamine therapy, with elevated AChR antibodies [7]. After stopping D‑penicillamine and starting short‑term pyridostigmine, ocular symptoms resolved within days, and at the six‑month follow‑up, he was symptom‑free without any MG treatment [7]. That rapid, complete recovery after drug withdrawal alone is characteristic of classic D‑penicillamine‑induced MG [7]. In contrast, our patient required prolonged immunosuppression (prednisone for 12 weeks) and remains on pyridostigmine at 12 months, indicating that SGLT‑2 inhibitor‑unmasked MG may be more persistent [7]. Moreover, our patient had histologically proven thymic follicular hyperplasia, a persistent autoimmune substrate, whereas the D‑penicillamine case lacked identifiable thymic pathology [7].

Checkpoint inhibitor-related MG represents the closest parallel to our case [8]. A large 2026 pharmacovigilance analysis of the FAERS identified 26,629 neurological immune‑related adverse events associated with ICIs, with MG being one of the most frequently reported categories (male predominance: 53.5%; median age: 67 years) [8]. The median time to onset was 30 days (approximately four weeks), which is similar to the eight‑week latency in our patient [8]. Moreover, 91.2% of these reports were classified as serious, highlighting the potentially life‑threatening nature of ICI‑induced neuromuscular complications [8].

MG induced by beta‑blockers is rare [9]. Sharifkazemi and Ziya reported a case of sotalol‑induced generalized and ocular MG in a patient with atrial fibrillation [9]. The patient developed bilateral ptosis, diplopia, dysphagia, and proximal limb weakness approximately three weeks after starting sotalol [9]. The diagnosis was confirmed by a positive neostigmine test and elevated anti‑AChR antibodies [9]. After sotalol was discontinued, symptoms improved gradually, but the patient still required pyridostigmine for several months [9]. This case differs from ours in several ways [9]. First, the latency was shorter (three weeks vs. eight weeks) [9]. Second, the patient had generalized weakness involving limbs, whereas our patient's weakness remained strictly ocular and bulbar [9]. Third, although sotalol withdrawal led to improvement, the patient still needed ongoing pyridostigmine, similar to our case [9]. However, in contrast to our patient, no thymic pathology was reported, and the patient did not require corticosteroids for remission [9].

The uniqueness of our case in relation to drug-induced MG results from three main considerations. Firstly, the new type of medication involved is one that has never been documented to cause such a condition before, namely, SGLT-2 inhibitors. Secondly, the diagnosis of thymic follicular hyperplasia has been confirmed via histopathology, thereby confirming the underlying immunologic mechanism. Thirdly, cessation of the medication was not enough by itself to lead to remission, hence the need for conventional treatment for MG.

The mechanism by which an SGLT-2 inhibitor might unmask subclinical MG is hypothesized based on established immunology, though causality remains unproven. Lee and Riella comprehensively reviewed the immunomodulatory and anti‑inflammatory effects of SGLT‑2 inhibitors, noting that these agents influence immune cell metabolism, reduce pro‑inflammatory cytokine production, and modulate T‑cell differentiation [10]. Importantly, they highlight that SGLT‑2 inhibitors can attenuate NF‑κB signaling and the NLRP3 inflammasome while shifting macrophage polarization from a pro‑inflammatory M1 toward an anti‑inflammatory M2 phenotype [10]. In a patient with pre‑existing thymic follicular hyperplasia and subclinical AChR antibody production, both established predisposing factors for spontaneous MG, the addition of empagliflozin may have acted as a potential trigger or unmasking factor rather than a direct causative agent. Thus, our findings should be interpreted as hypothesis‑generating; confirmation of a causal relationship requires further pharmacovigilance and mechanistic studies [10]. In the context of a patient with pre‑existing thymic follicular hyperplasia, which serves as a reservoir for autoreactive AChR‑specific B cells within germinal centers, the net immunomodulatory effect of an SGLT‑2 inhibitor may paradoxically undermine established immune tolerance. This disturbance could allow clonal expansion of autoreactive B cells, increased production of pathogenic AChR antibodies, and their trafficking to the neuromuscular junction, leading to clinical disease. This "second hit" paradigm is well recognized in autoimmunity and has been invoked to explain ICI‑associated MG [10]. Our patient's eight‑week latency period is consistent with the time required for these immunologic events to reach the clinical threshold.

This being only one report, we cannot prove the causality of the event; thus, the spontaneous onset of MG following the introduction of the drug is still possible. A Naranjo criteria score of 6 points can classify the occurrence as "probably an adverse effect" but not as "definitely an adverse effect" [4]. Furthermore, there was no attempt at a rechallenge with empagliflozin owing to ethical issues, as it might have resulted in a myasthenic crisis, which could be morbid and fatal in nature. We did not carry out tests, including T-cell subpopulation assessment, cytokine assay, and AChR Ab epitope specificity analysis, that could help in demonstrating the direct effect of the drug in this patient. Moreover, the presence of thymic hyperplasia in the patient would eventually result in MG, whether he were on empagliflozin or not, although the absence of the disease for several years negates the possibility. Finally, due to being a single patient report, there can be no calculation of the absolute risk of MG caused by SGLT-2 inhibitors.

Nevertheless, the temporal relationship between drug initiation and symptom onset, lack of other inciting factors, successful dechallenge, in-depth investigations, and histological confirmation make it worthwhile to publish this unique case as the first-ever documented case of MG associated with SGLT-2 inhibitors. Physicians using SGLT-2 inhibitors for the management of diabetes mellitus must understand that any patient with sudden-onset intermittent ptosis, double vision, speech impairment, and/or difficulty swallowing requires further investigations as a newly diagnosed MG patient. The ice pack test, which entails applying an ice pack on the affected lid for two minutes with at least 2 mm improvement, is still the easiest bedside test with high specificity for MG [3]. When MG is suspected, early referral to a neurologist and an anti-AChR antibody test must follow. In cases where MG is confirmed, apart from stopping the offending drug, immunosuppressive therapy for MG, including pyridostigmine and, when needed, steroids, must follow.

## Conclusions

This is the first case report of MG discovered in a patient receiving an SGLT-2 inhibitor (empagliflozin). The patient had incidental thymic enlargement on a computed tomography scan, later confirmed as follicular hyperplasia by pathology. Symptoms developed eight weeks after the initiation of the agent, with no other identified cause. A positive dechallenge and confirmatory results yielded a Naranjo score of 6, indicating a probable adverse drug reaction. While causality remains unproven, this case raises a potential signal that SGLT-2 inhibitors may unmask subclinical MG in susceptible individuals. Therefore, in patients with diabetes mellitus who develop new ocular or bulbar symptoms while on SGLT-2 inhibitors, MG should be considered, and further pharmacovigilance studies are warranted to clarify this association. Pharmacological remission was achieved during 12 months of follow-up.
